# Supplementary material for: Analysis of the senescence secretome during zebrafish retina regeneration
Source: Front Aging. 2025 Apr 16;6:1569422. doi: 10.3389/fragi.2025.1569422 (PMC12040975; doi:10.3389/fragi.2025.1569422)
Supplement: Supplementary file 5 [file DataSheet1.pdf]

Supplemental Figures for Konar et al, 2025.

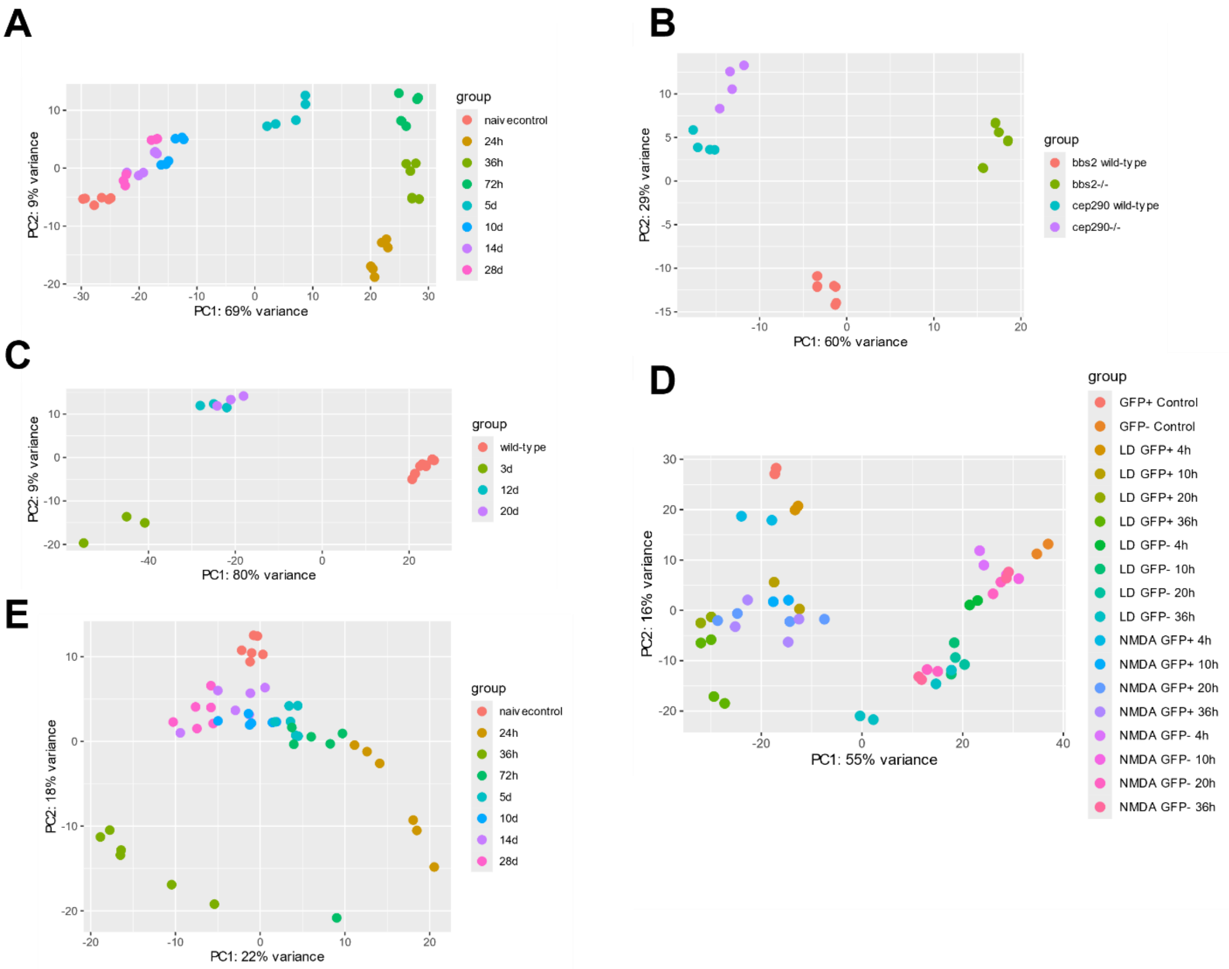

Supplemental Figure 1. Principal component analysis of bulk RNAseq datasets. A) Kramer et al. 2021, B) Grabinski et al. 2023, C) Konar et al. 2025 (this publication), D) Hoang et al. 2020, E) Kramer et al. 2023

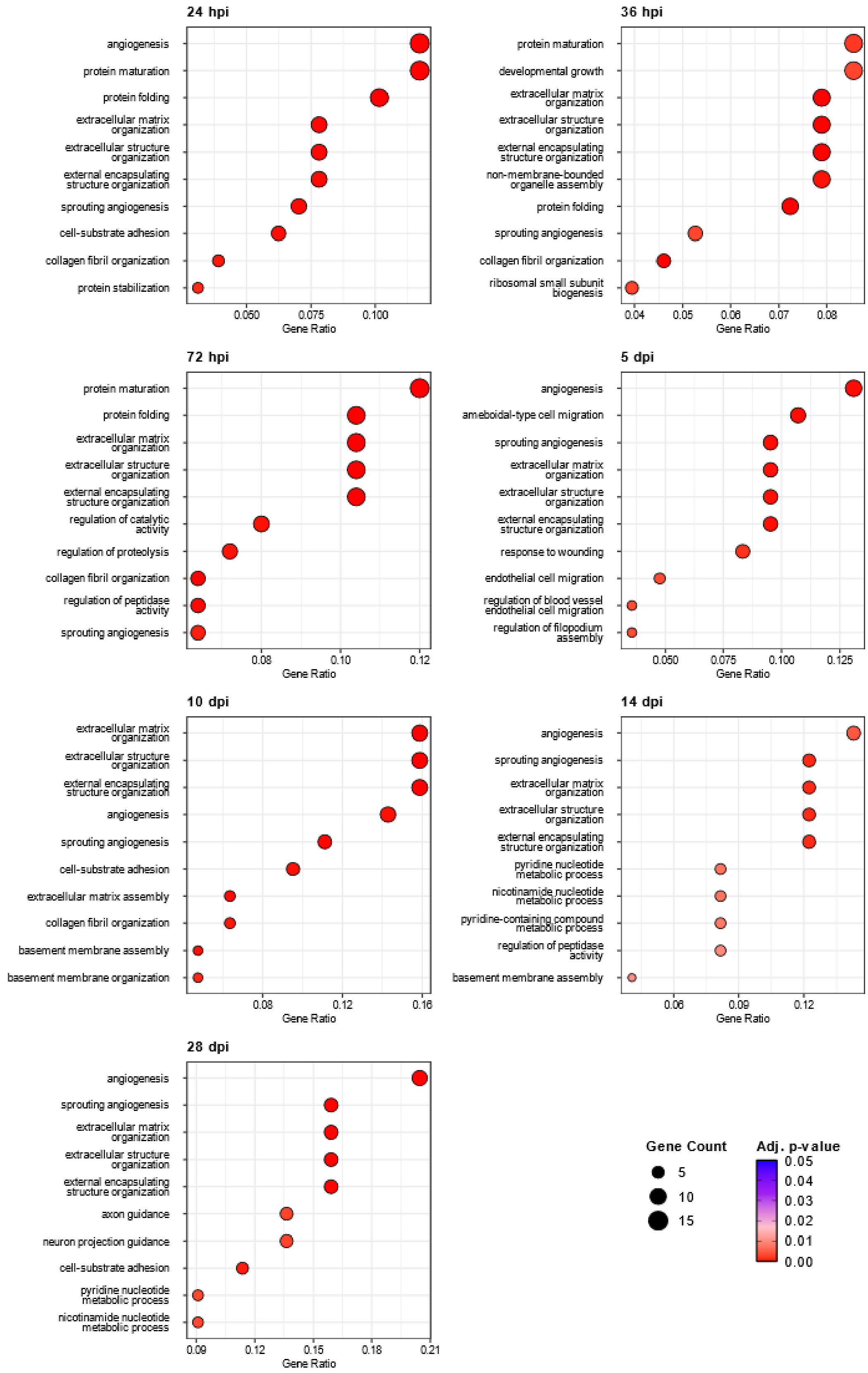

24 hpi
angiogenesis
protein maturation
protein folding
extracellular matrix organization
extracellular structure organization
external encapsulating structure organization
sprouting angiogenesis
cell-substrate adhesion
collagen fibril organization
protein stabilization
0.050
0.075
0.100
Gene Ratio
36 hpi
protein maturation
developmental growth
extracellular matrix organization
extracellular structure organization
external encapsulating structure organization
non-membrane-bounded organelle assembly
protein folding
sprouting angiogenesis
collagen fibril organization
ribosomal small subunit biogenesis
0.04
0.05
0.06
0.07
0.08
Gene Ratio
72 hpi
protein maturation
protein folding
extracellular matrix organization
extracellular structure organization
external encapsulating structure organization
regulation of catalytic activity
regulation of proteolysis
collagen fibril organization
regulation of peptidase activity
sprouting angiogenesis
0.08
0.10
0.12
Gene Ratio
5 dpi
angiogenesis
ameboidal-type cell migration
sprouting angiogenesis
extracellular matrix organization
extracellular structure organization
external encapsulating structure organization
response to wounding
endothelial cell migration
regulation of blood vessel endothelial cell migration
regulation of filopodium assembly
0.050
0.075
0.100
0.125
Gene Ratio
10 dpi
extracellular matrix organization
extracellular structure organization
external encapsulating structure organization
angiogenesis
sprouting angiogenesis
cell-substrate adhesion
extracellular matrix assembly
collagen fibril organization
basement membrane assembly
basement membrane organization
0.08
0.12
0.16
Gene Ratio
14 dpi
angiogenesis
sprouting angiogenesis
extracellular matrix organization
extracellular structure organization
external encapsulating structure organization
pyridine nucleotide metabolic process
nicotinamide nucleotide metabolic process
pyridine-containing compound metabolic process
regulation of peptidase activity
basement membrane assembly
0.06
0.09
0.12
Gene Ratio
28 dpi
angiogenesis
sprouting angiogenesis
extracellular matrix organization
extracellular structure organization
external encapsulating structure organization
axon guidance
neuron projection guidance
cell-substrate adhesion
pyridine nucleotide metabolic process
nicotinamide nucleotide metabolic process
0.09
0.12
0.15
0.18
0.21
Gene Ratio
Gene Count
5
10
15
Adj. p-value
0.05
0.04
0.03
0.02
0.01
0.00

Supplemental Figure 2. Biological process Gene Ontology for enriched SASP factors after acute light damage. Bulk RNAseq was performed on whole zebrafish retinas after acute light damage and compared to undamaged controls. For each time point, Gene Ontology enrichment for biological process was conducted for all SASP factors and markers enriched beyond a log2FC of 1 with adjusted p-values < 0.05 using clusterProfiler. The top 10 enriched terms for the respective time points are displayed as dot plots.

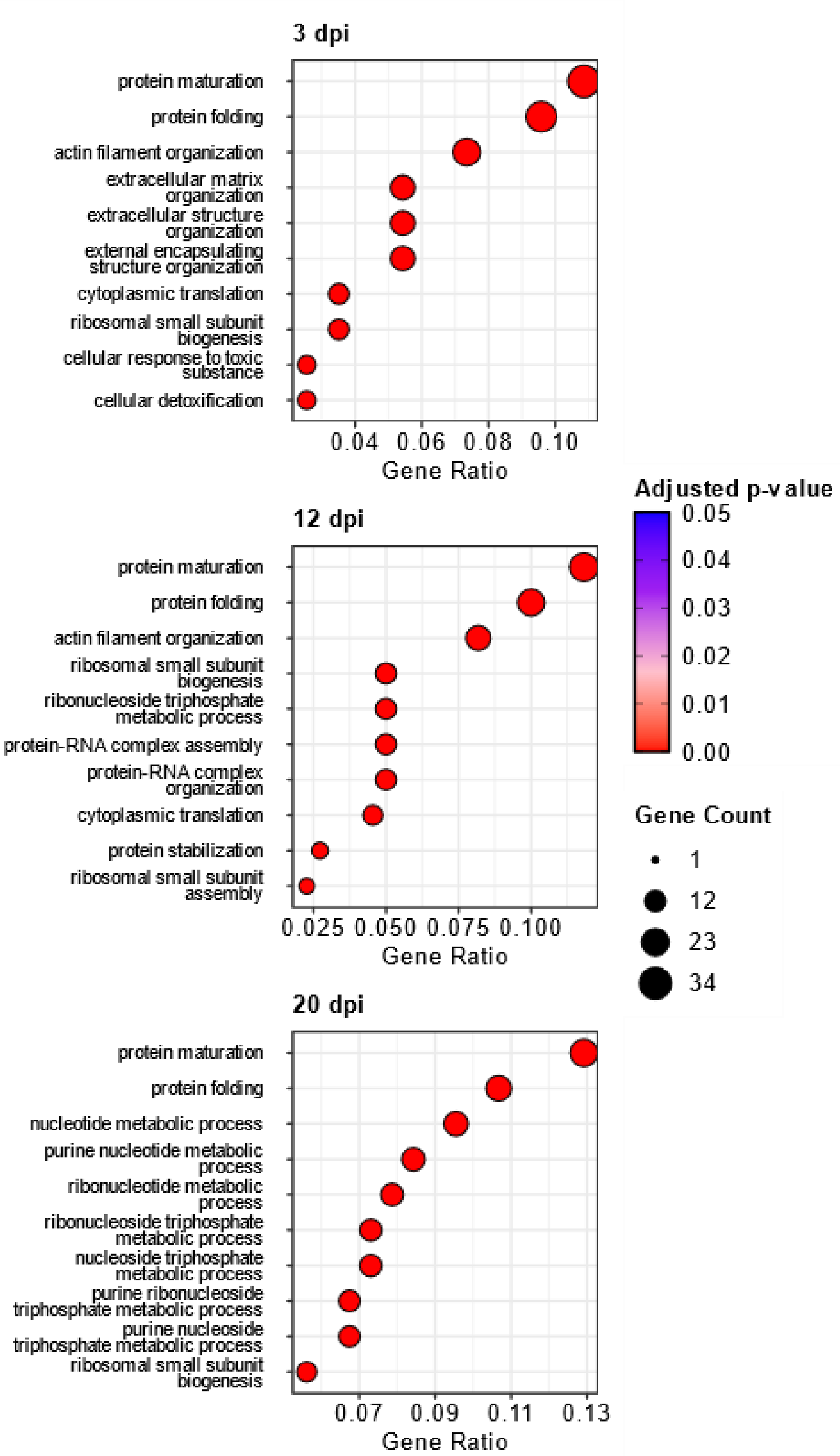

Supplemental Figure 3. Biological process Gene Ontology for enriched SASP factors after NMDA damage. Bulk RNAseq was performed on whole zebrafish retinas after intravitreal injections of NMDA and compared undamaged controls (Grabinski, Parsana et al. 2023). For

each time point, Gene Ontology enrichment for biological process was conducted for all SASP factors and markers enriched beyond a log2FC of 1 with adjusted p-values < 0.05 using clusterProfiler. The top 10 enriched terms for the respective time points are displayed as dot plots.

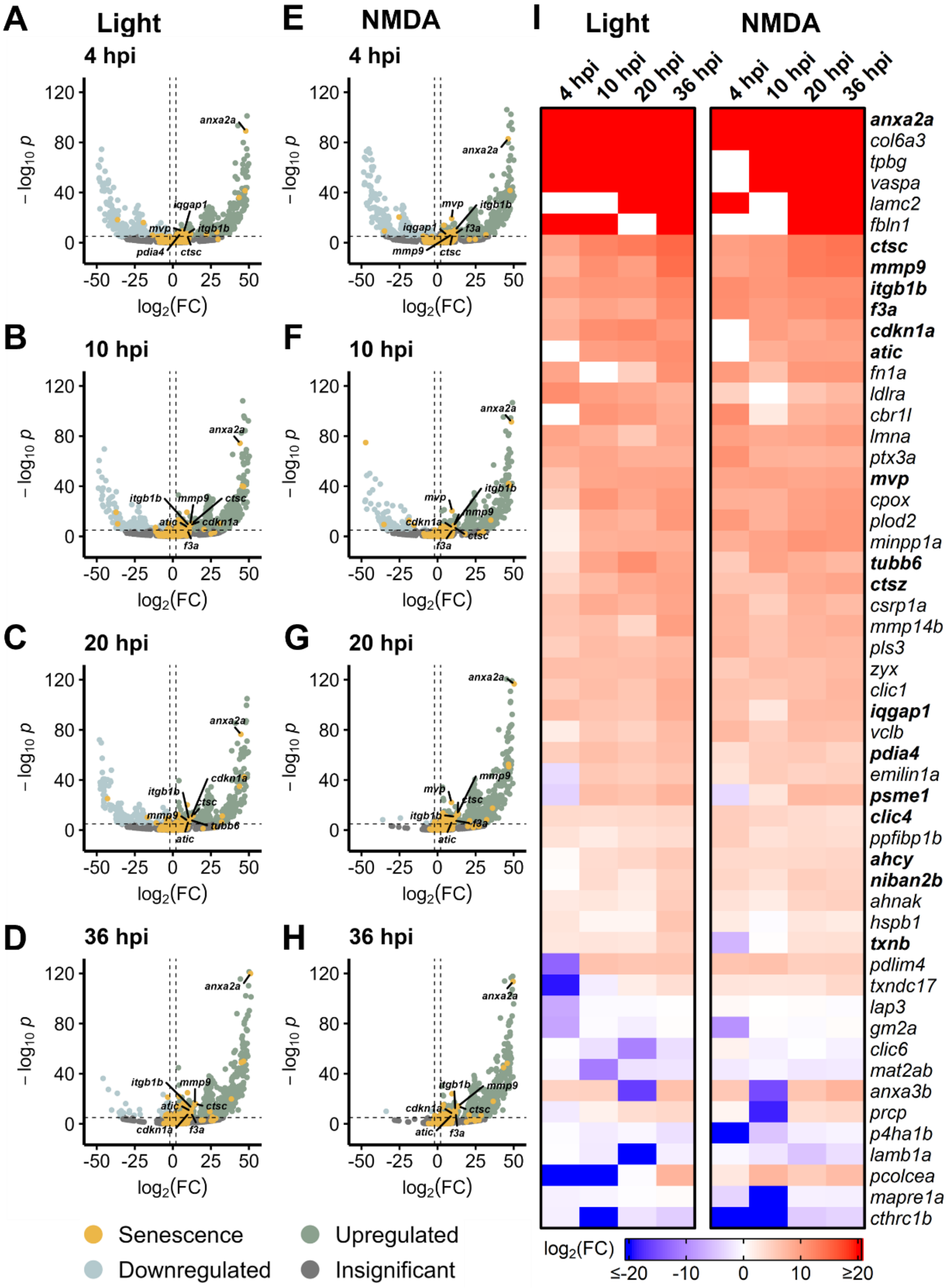

Supplemental Figure 4. SASP factor conservation across acute damage models in non-Müller glia. Bulk RNAseq was performed on non-Müller glia after either NMDA or light damage compared to undamaged controls. (A-D) Volcano plots showing differentially expressed SASP

factors at the indicated times post light damage. (E-H) Volcano plots showing differentially expressed SASP factors at the indicated times post NMDA damage. Dashed lines represent values with a |log2FC| > 2 and p-values < 10-6. Select up-regulated factors are labeled for each time point. (I) Heatmap showing enrichment of SASP factors at the indicated times post light or NMDA damage. SASP factors were included in the heatmap if they had a log2FC > 5 with p-values < 10-6 for at least one time point; bolded genes are part of the SRRF.

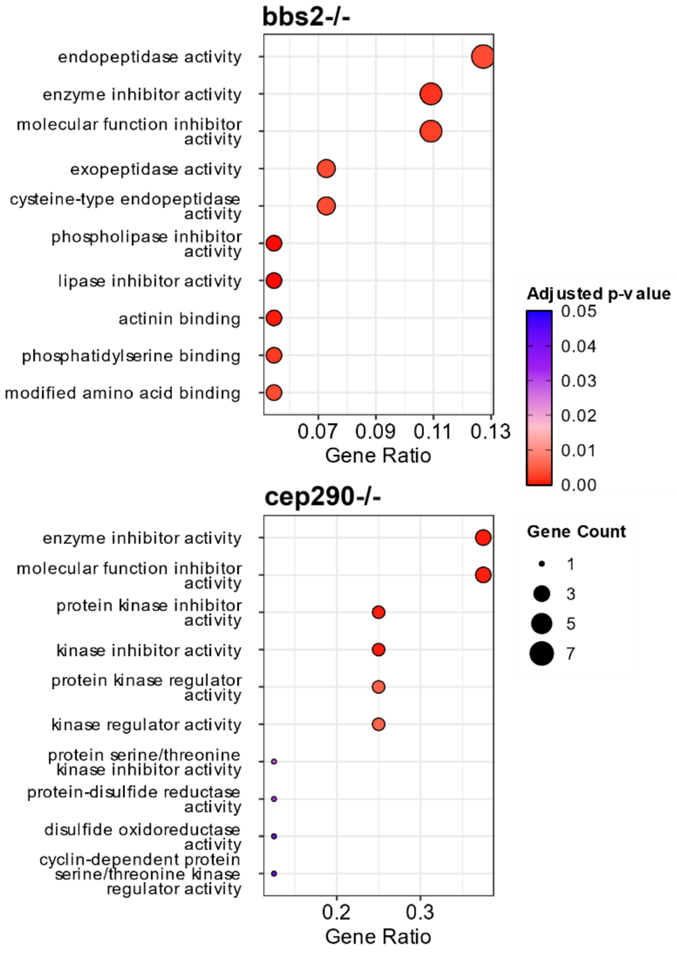

Supplemental Figure 5. Molecular Function Gene Ontology for enriched SASP factors in genetic mutants with chronic photoreceptor damage. Bulk RNAseq was performed on whole zebrafish retinas from bbs2-/- and cep290-/- ciliopathy mutants. For each genetic mutant, Gene Ontology enrichment for molecular function was conducted for all SASP factors and markers enriched beyond a log2FC of 1 with adjusted p-values < 0.05 using clusterProfiler. The top 10 enriched terms for the respective time points are displayed as dot plots.

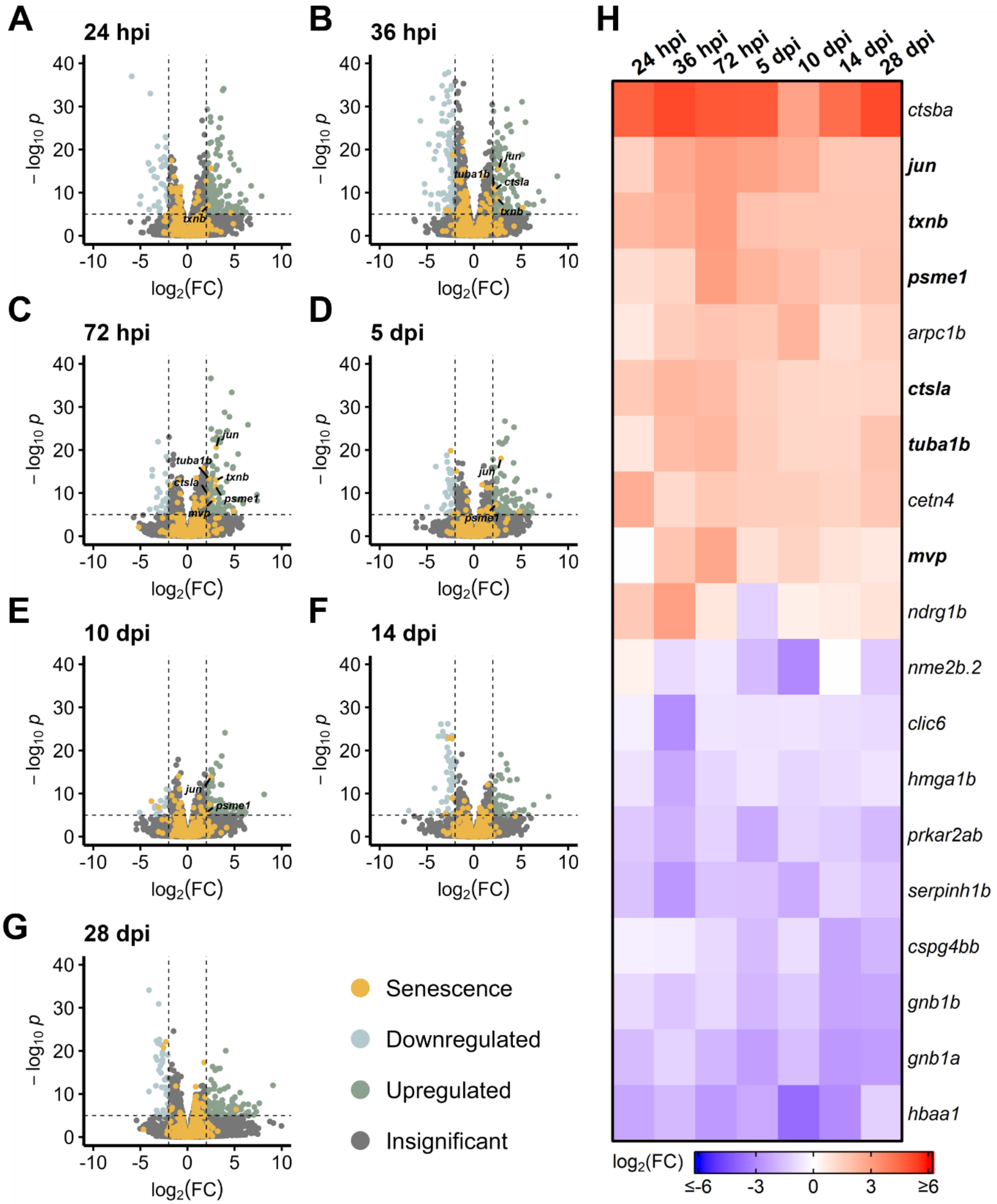

Supplemental Figure 6. Differentially expressed SASP factors after chronic light damage. Bulk RNAseq was performed on whole zebrafish retinas after chronic light damage. (A-G) Volcano plots depicting up- and down-regulated SASP factors and senescence markers at the

indicated times post damage. Dashed lines represent values with a |log2FC| > 2 and p-values < 10^{-6}. Select factors are labeled for each time point. (H) Heatmap showing enrichment of SASP factors at the indicated times post chronic light damage. SASP factors and senescence markers were included in the heatmap if they had a log2FC > 2 with p-values < 10^{-6} for at least one time point; bolded genes are part of the SRRF.
